# Fractures of the Spine

**Published:** 1861-08

**Authors:** G. K. Amerman

**Affiliations:** Chicago, Ill.


ARTICLE V.

### FRACTURES OF THE SPINE.

At the last regular meeting of the Chicago Medical Society, Dr. Amerman presented two specimens of fractures of vertebræ, with the following histories:

Case I. Henry Aurz, aged 27, born in Germany; by occupation a lumberman; of good health and temperate habits.

On the 16th of March, 1861, whilst engaged in log-rolling, his foot slipped, and he fell in such a manner that several logs rolled on him, bending his body forward. He was immediately deprived of motion and sensation in his lower limbs. April 1st, fifteen days after the occurrence of the injury, he was admitted into the Chicago City Hospital. At the time of his admission the paralysis was complete. The urine and fæces were passed involuntarily. On examining the spine, an irregularity was readily perceived in the lower dorsal region, but no crepitus. There was also some pain on pressure, otherwise the parts appeared natural. A small bed-sore existed over the sacrum, produced previous to his removal to the hospital. The urine was thick, and deposited a heavy sediment of mucus, pus and salts. Pulse ranged from 100 to 120; appetite good; no pain; and patient rested well at night.

The treatment consisted in placing the patient in the most favorable position to guard against pressure and the occurrence of bed sores, and paying the strictest attention to cleanliness. In addition he was given good diet, quin et ferri, and emulcent drinks. He died April 12, twenty-seven days after the receipt of the injury. Autopsy held day after death. The soft parts were healthy, except just around the seat of the injury, where there were some ecchymosed spots. The injuries done to the vertebræ were confined to the eleventh and twelfth dorsal. The only injury to the eleventh was fracture of the right transverse process, at its junction with the lamina and pedicle. The injury of the twelfth consisted of a fracture of the body near its upper surface, extending on the left side through the pedicle, between the superior articular and transverse processes, thus allowing the eleventh to project forward and compress the cord. The left transverse process was also entirely broken off and lost in preparing the specimen. The cord was uninjured.

Case II. John Donnally, aged 28; by occupation a carpenter; general health good; habits temperate. On the 10th of March, 1861, a large piece of timber struck him a severe blow in the back, and he fell about twelve feet. He was immediately paralyzed in his lower extremities. On the 24th of April, six weeks after the injury, he was brought to the Chicago City Hospital. At the time of his admission there was complete loss of sensation and motion of the lower half of the body with involuntary discharge of the urine and fæces, and an immense sloughing bed-sore over the sacrum. The treatment consisted in removing pressure as much as possible, keeping the patient clean, allowing full diet, and other measures of a supporting character. He died May 24th, one month after admission into the Hospital, and eleven weeks after the receipt of the injury. Autopsy day after death. No injury of external soft parts. The injuries of the vertebræ were of the third and fourth dorsal. The fourth was fractured entirely through the body, near its middle, with the upper fragment pushed forward and toward the left side. No appearance of any attempt at union. The third was the seat of a very interesting condition. The body had sustained an impacted fracture, the lower half being driven into the upper, and ossific material poured out in considerable quantity, filling up the irregularities. The right transverse process had been entirely broken off, but re-united as firmly as ever with slight displacement downward. The cord was softened for about two inches of its length, and compressed.

---

These cases are highly suggestive to a thoughtful surgeon. We do not think that all the possibilities of surgical help are exhausted in the treatment, or rather non-treatment of fractured vertebræ. As these cases came under the care of Dr. Amerman respectively two and six weeks after the receipt of the injuries, it was obviously too late for him to propose any radical measures; but where a case is brought to the surgeon immediately after the accident, it seems to us that more might often be done than our text books allow us to undertake.

In Case I, had the surgeon been fortunate enough to see it at the outset, there were obviously two possible modes of relieving the spinal cord of pressure. The first would have been by a firm and steady extension upon the head and hips, as has been advised in cervical fractures. By this method, possibly, the eleventh vertebra might have been reduced to its position. If this effort failed, the next resource would be to saw away the compressing arches of the tenth and eleventh vertebræ. This is absurdly objected to by authors, on the ground that it would lay open the dura mater and evacuate the spinal fluid; but no such result need follow. It is not much more difficult to cut away the arches of the vertebræ without wounding the dura mater, than it is to trephine the skull with the same immunity. The same reasons for the operation exist in both cases, and there is no ground for expecting more danger in one than the other. Total compression of the spinal cord is a mortal injury, unless relieved, and hence it is foolish to object to any moderately dangerous operation which promises possible success. It would be quite as rational to reject trepanning in depressed fractures of the skull.

Probably a certain number of failures would result from the fact that sometimes the cord is completely crushed off and disorganized, and in others it is so strained across the displaced fragments of the body of the vertebra, as to receive effectual compression from the front, but a good many cases would probably remain, which would justify the surgeon's boldness by a successful issue.

---

Diuretic Action of Colchicum.—Prof. Wm. A. Hammond, of the University of Maryland, in some experiments instituted upon himself and others, arrived at the following conclusions: 1st, That the colchicum increases the quantity of urine; 2d, That it increases the total amount of solid matters eliminated; 3d, That this increase is mainly due to an augmentation of the organic matter; 4th, That the amount of uric acid does not appear to be affected.

He thinks colchicum must be allowed to be a true depurator of the blood, and that hence we have an explanation of its good effects in those blood diseases, gout and rheumatism.

It seems that no constant effect was produced upon the quantity of uric acid eliminated, and hence these experiments do not conflict with those of Dr. Garrod. We are not, however, bound to admit that the presence of uric acid in the blood in increased amount during a paroxysm of gout or rheumatism, is the cause of that paroxysm; and, consequently, because colchicum does not increase the quantity of this substance found in the urine, we are not to suppose that the remedy in question does not exert its influence through the kidneys.—*American Medical Monthly.*

